# A long-term dataset on wild bee abundance in Mid-Atlantic United States

**DOI:** 10.1038/s41597-020-00577-0

**Published:** 2020-07-20

**Authors:** Melanie Kammerer, John F. Tooker, Christina M. Grozinger

**Affiliations:** 1 Intercollege Graduate Degree Program in Ecology, Pennsylvania State University, University Park, PA USA; 2 Department of Entomology, Center for Pollinator Research, Huck Institutes of the Life Sciences, Pennsylvania State University, University Park, PA USA

**Keywords:** Conservation biology, Biodiversity, Entomology

## Abstract

With documented global declines in insects, including wild bees, there has been increasing interest in developing and expanding insect monitoring programs. Our objective here was to organize, validate, and share an analysis-ready version of one of the few existing long-term monitoring datasets for wild bees in the United States. Since 1999, the Native Bee Inventory and Monitoring Lab (BIML) of the United States Geological Survey has sampled wild-bee communities in the Mid-Atlantic U.S., but samples were collected in multiple studies and the datasets are not fully integrated. Furthermore, critical information about sampling methodology was often lacking, though these factors can significantly influence collection outcomes and must be considered in analyses. We cleaned and verified BIML data from Maryland, Delaware, and Washington DC, USA, and generated sampling methodology for over 84% of the 99,053 pan-trapped occurrences in this region. We enthusiastically invite creative analyses of this rich dataset to advance understanding of the biology and ecology of wild bees, inform conservation efforts, and perhaps help design a nationwide bee monitoring program.

## Abstract (summary)

**Table:** 

Measurement(s)	Abundance
Technology Type(s)	digital curation
Factor Type(s)	site • year
Sample Characteristic - Organism	Apoidea
Sample Characteristic - Location	United States of America

Machine-accessible metadata file describing the reported data: 10.6084/m9.figshare.12473270

## Background & Summary

Wild bees are crucial pollinators of many crop and wild-plant species. Globally, 87 out of 115 major food crops require insect pollination and 78% of temperate plant species and 94% of tropical plant species require insect pollination for reproductive success and persistence^1,2^. Wild-bee conservation is crucial for global biodiversity as pollinators represent several extremely diverse taxa and have a vital co-evolved role in supporting and selecting for diverse plant communities^3^.

In Europe and the United States, there has been a surge of interest in developing programs to monitor wild-bee populations^4–7^. There have been documented declines in several North American bumble bees populations and species^8^, but we largely lack baseline data to assess declines in many other taxa. In response to heavy colony losses since 2006, monitoring of honey bee (*Apis mellifera L*.) populations has greatly increased^9^. These data have greatly contributed to our understanding of honey bee population dynamics, ecological interactions, colony stressors, and effective management.

We expect coordinated, wild-bee monitoring will enable similar advances in understanding wild-bee diversity, natural history, and conservation strategies, but there are still several challenges in collecting and analysing monitoring data. Ideally, a wild-bee monitoring program, or any monitoring program for that matter, would utilize consistent methods over time to detect long-term population trends. In practice, monitoring protocols often shift over time, and many existing datasets were compiled from studies using multiple sampling methods^10^.

Our goal here was to organize, validate, and share an analysis-ready version of one of the few existing long-term, wild-bee monitoring datasets in the United States. Since 1999, the Native Bee Inventory and Monitoring Lab (BIML) of the United States Geological Survey has collected, curated, and identified over 99,000 specimens of more than 314 wild bee species from over 1400 sites in Maryland, Delaware, and Washington DC. The BIML collection is among the most geographically dense, recent sampling of wild bees in the country. Also, all specimens were lethally collected and expert-identified to species, which provides a crucial comparison for observation-based datasets with coarser taxonomic information, including rapidly growing citizen science data^11,12^.

BIML data contain considerable diversity in wild-bee-sampling methods and critical sampling information is often missing or not easily accessible to end-users. One goal of BIML research was to test a variety of passive sampling methods to establish a standard protocol for wild-bee monitoring. They studied sampling efficiency of passive pan traps (bee bowls) with varying trap color, volume, and liquid. Pan traps are a reliable method for monitoring wild bees^13,14^, particularly in combination with net collections or transect walks, but the specific pan-trapping method, especially trap color and visibility, influences the abundance and composition of wild bees collected^15–17^. To fully utilize the BIML dataset collected with multiple methods, trap color should be included as a variable in statistical analyses. Currently, this is not possible with the publicly available BIML data^18^ as 90% of the pan-trapped occurrences in Maryland, Delaware, and DC have no trap color data. Similarly, 36% of occurrences are missing trap volume and 21% lack sampling effort (the number of traps used during each sampling period). Fortunately, much of the missing information was recorded in BIML field notes, but not in a consistent, accessible format.

To fill these gaps, we cleaned and verified BIML data and extracted trap color, volume, and sampling effort for more than 84% of the pan-trapped occurrences. We focused on occurrences collected in pan traps because they represent more than 82% of the larger BIML dataset. We also created, for all pan-trapped occurrences, identifier variables and verified species binomials, date, and locality. We enthusiastically invite analyses of this rich dataset to advance understanding of bee biology and ecology, inform current wild bee conservation efforts, and perhaps even help design a national wild bee monitoring program.

## Methods

### Identifier variables

To facilitate aggregation and analysis of the BIML data, we added ‘site’, ‘site-year’, ‘sampling event’, and ‘transect’ identifier variables. We defined ‘sites’ as unique combinations of latitude and longitude, and ‘site-years’ as unique combinations of site and year of sampling. Within site-years, we defined ‘sampling events’ according to the date of sampling and ‘transects’ as unique combinations of sampling event and text field notes. For some specimens, field notes included a transect ID, indicating that the BIML used multiple sets of pan traps at the same site. In other cases, field notes recorded differing sampling methods, or different information on the number of missing traps (traps that were cracked, tipped over, or otherwise compromised). If field notes recorded different methods or number of lost traps, we assumed that the BIML deployed multiple sets of traps (transects). We reviewed the field notes for all sampling events with multiple transects and reassigned these occurrences to a single transect if there was no evidence of multiple transects in the field notes.

### Locality and taxonomic identification

**Fig. 1:**
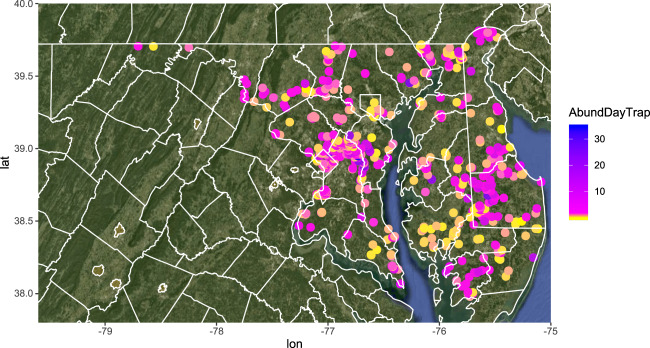
Abundance per day per trap of wild bees at locations surveyed between 2002 and 2016 by the United States Geological Survey Native Bee Inventory and Monitoring Lab (USGS BIML).

Next, we reviewed and excluded occurrences lacking critical date and locality information. We removed all occurrences lacking sampling date or latitude and longitude of sampling location and occurrences with duplicated specimen identifiers. We filtered occurrences to a limited geographic area (Maryland, Delaware, and Washington DC, Fig. 1) that represents the densest region of BIML sampling (39.6% of dataset). This filtering removed wild-bee communities collected in desert or tropical biomes, which are likely governed by very different floral resource and climate dynamics^19,20^, and within the Mid-Atlantic USA, limited sampling locations to a region with a consistent dominant forest type^21^. Bee occurrences in 1999 and 2001 represented fewer than three sites per year, so we removed these years, retaining sites sampled from 2002–2016.

We also filtered data to our taxa of interest. We removed non-bee occurrences (species outside superfamily *Apoidea*, clade *Anthophila*) and records lacking species-level identity, discarding occurrences identified to family or genus (Online-only Table 1). Almost all non-bee occurrences we removed were wasps in the *Vespidae*, *Crabronidae*, and *Sphecidae* families, which are primarily predators, rather than pollen-collectors like most wild bees. For the transect-level dataset^22^ (see ‘Data Records’ below), we calculated the abundance of *Apis mellifera L*., then removed *A. mellifera* from the dataset before calculating total bee abundance per transect, since often *A. mellifera* specimens likely originated from managed colonies and are not considered to be wild bees.

We verified species names by cross-referencing all species binomials with the Discover Life database^23^. We corrected genus and species names that were clear spelling errors (Online-only Table 1) and consulted the original data source (S. Droege) for remaining species binomials that did not exist on Discover Life. We also referenced Discover Life occurrence maps to confirm that all species in the BIML dataset occur in the Mid-Atlantic US. After these data cleaning steps, we removed six occurrences of the remaining five unknown or out-of-region species (Online-only Table 1). Some species in the BIML data were identified singularly and as part of a species set. To avoid double counting these species, we created a new variable with cleaned, mutually exclusive species names (termed ‘grouped name’). In ‘grouped name’, we combined singular species names with their associated species sets (Online-only Table 1). For example, we reclassified occurrences identified as *Halictus ligatus/poeyi, Halictus ligatus, or Halictus poeyi* to *Halictus ligatus/poeyi* to avoid inflating future species richness estimates when occurrences might be the same species. In the final occurrence datasets, we included the cleaned, singular names (‘name’) and cleaned, grouped names (‘grouped_name’), so future analysts can select the appropriate taxonomic aggregation for their research objectives. Voucher specimens for most species in the BIML dataset are housed in the Smithsonian collection, but some are not yet permanently archived. We suggest interested parties contact Sam Droege (current email: sdroege@usgs.gov) to access voucher specimens. We also included, to the best of our knowledge, current affiliations for individuals who identified BIML specimens (Supplementary Information, Table S1), and standardized names of identifiers (‘identifiedBy’) in the final datasets.

### Sampling method and effort

To describe sampling method and effort, we used regular expressions to extract these data from field notes. We sought to compare bee communities sampled with a standard methodology, so we discarded bee occurrences collected with vane traps or nets, only retaining occurrences sampled with pan traps (i.e., bee bowls). Using the *stringr* package in R^24,25^, we searched the text of field notes to document trap volume, trap color, total number of traps, and the number of traps missing or disturbed. The most common BIML pan-trapping method involved setting out traps of multiple colors and combining the bees in all traps into one sample. Consequently, BIML recorded trap color in field notes as the number of traps of each color used for a specific sampling event. We designed regular expressions to extract the number of traps for the eight most common colors (white, blue, yellow, pale blue, fluorescent yellow, fluorescent blue, and florescent pale blue). For some occurrences, our regular expressions yielded no sampling information, so we manually reviewed these field notes and recorded any data missed by the automated search.

Next, we simplified the trap color and volume classification to facilitate future statistical analyses. To reduce the number of trap volume categories, we rounded trap volume to the nearest 0.5 ounces, and removed trap volumes greater than 40 ounces, assuming these were errors in data entry or extraction. When the trap color or volume used at a specific site changed within a year, we manually reviewed the field notes and corrected color or volume classifications when necessary. After correcting these discrepancies, we found the BIML very rarely changed sampling methods within a year, so we filled in most missing trap color or volume information by assuming a constant sampling method for all transects within a site-year. Finally, we combined rarely used color/volumes (fewer than 1% of transects) into an ‘Other’ category. In the archived datasets with sampling information, we included original and simplified variables for trap color and volume.

Lastly, we summarized sampling effort and calculated effort-adjusted abundance of wild bees. We calculated the total number of traps for each sampling event, and, when available, we also described the number of traps missing or disturbed. If there was no documentation of missing or disturbed traps, we assumed all traps were recovered successfully. When the total number of traps differed between the field notes and ‘number of traps’ column, we selected the lower value. We defined the final number of traps in each transect as the original number minus the number missing or disturbed. We calculated the duration of sampling as the difference between the date traps were collected and date traps were set. If traps were set and collected on the same day, we set the duration of sampling to one day. For each occurrence in the BIML dataset, we converted bee abundance to abundance day^−1^ trap^−1^. We conducted all data manipulation and aggregation with the R statistical and computing language 3.6.0^25,26^

## Data Records

We produced two primary datasets and a third analytical dataset^22^ (details below), which are all archived at figshare.com. The first dataset (‘Mid-Atlantic USA wild bee occurrences, all records’) contains all 99,053 wild bee occurrences collected in pan traps by BIML from 2002 to 2016 in Maryland, Delaware, and Washington DC, USA. We describe all variables included in dataset one in Table 1. The second dataset (‘Mid-Atlantic USA wild bee occurrences, records with sampling info’) is all pan-trapped occurrences with sampling effort information. We retained 83,583 wild bee occurrences in dataset two representing 300 species from 1225 sites sampled over 1322 site-years. Dataset two documents the trap color and volume when these data were available. See Online-only Table 2 for full meta-data for dataset two.

**Table 1:** Description of all fields in dataset 1, all wild bee specimens in Maryland, Delaware, and Washington DC. Variables marked as ‘Original’ are unmodified from Droege and Sellers^18^.

Field Name	Unit	Description
Identifiers		
identifier		Original, specimen ID
id		Original, specimen URL
TransectID		transect ID
SamplEvent		sampling event ID
SiteID		site ID
SiteID_Year		site ID and year of bee sampling
year		year of bee sampling
Taxonomy		
name		taxonomic classification
Genus		taxonomic classification
species		taxonomic classification
grouped_name		grouped taxonomic classification
orig_name		Original, taxonomic classification
sex		Original, specimen sex
identifiedBy		Individual who determined specimen identity
Locality and Date		
latitude	decimal degrees	Original
longitude	decimal degrees	Original
coordinateUncertaintyInMeters	meters	Original
time1		Original, start date and time
time2		Original, end date and time
startdate	month/day/year	sampling start date
enddate	month/day/year	sampling end date
country		Original
countryCode		Original
state		Original
county		Original
municipality		Original
Field notes		
habitat		Original, habitat in location of sampling
field_note		Original
note		Original
modif_fieldnote		modified field note used for automated string search
modif_note		modified note used for automated string search
Sampling method and effort		
SampleType		type of sampling method
TrapLiquid		Original, liquid used in traps
TrapColor		Original, color of trap
TrapVolume		Original, volume of trap
Ntraps		Original, number of traps
Wild bee abundance		
Abundance		wild bee abundance

We also generated an analytical dataset^22^ (‘Mid-Atlantic USA wild bee abundance per transect, records with sampling info’) derived from dataset two. Dataset three documents wild-bee abundance, trapping method, and sampling effort per transect (see ‘Identifier Variables’ methods above). At the transect level, we defined trap color and volume as the combination of all trap colors and volumes associated with specimens captured in a transect. Table 2 describes all meta-data for dataset three.

**Table 2:** Description of all fields in archived dataset 3, wild bee abundance per transect.

Field Name	Unit	Description
Identifiers		
TransectID		transect ID
SamplEvent		sampling event ID
SiteID_Year		site ID and year of bee sampling
*Apis mellifera* abundance		
apis_abund		*Apis mellifera* abundance
prop_apis		*Apis mellifera* proportional abundance
Sampling method and effort		
TstVolume		trap volumes used in this transect
TstColor		trap colors used in this transect
ColorVolume		color and volume of traps in transect
VolumeSimple		simplified classification of ‘TstVolume'
ColorSimple		simplified classification of ‘TstColor'
trapdays	days	length of sampling period
NTrapsFinal		number of successful traps (set-missing)
Wild bee abundance		
Abundance	specimens	wild bee abundance
AbundDayTrap	specimens day^−1^ trap^−1^	wild bee abundance day^−1^ trap^−1^

## Technical Validation

We verified our sampling method and effort data in two ways. First, as described above (see ‘Sampling method and effort’), we manually reviewed field notes for all occurrences lacking sampling effort information. This allowed us to correct instances where sampling data were missed by our automated processing. Second, for occurrences that had sampling effort information, we quantified the accuracy of our processing procedure. We selected a random sample of 1000 occurrences and compared field-note text and sampling-method variables from the original dataset with our sampling effort and method data. We noted whether trap color, trap volume, and the total number of traps were assigned correctly and quantified how much information we created compared with the original BIML dataset.

Overall, we were able to generate or verify pan-trap sampling effort and methodology for 84% of BIML specimens in the Mid-Atlantic U.S. We improved data coverage for trap color, trap volume, and sampling effort by 71%, 4.7%, and 6.1%, respectively. The percentage of occurrences with present, accurate sampling data was good to excellent for all three variables (trap color: 82.2%, trap volume: 88.6%, sampling effort: 95.9%,). Specifically, 888 observations in our validation set were missing trap color data. Using our automated text search, we accurately described trap color for 714 occurrences. Of the remaining 174 occurrences, we generated partial or incorrect data for 27 and 2 occurrences, respectively, and 145 had no color data in the text field notes. For trap volume and sampling effort (the total number of traps used in each sampling event), the original BIML dataset was much more complete. Out of 1000 specimens in our validation dataset, sampling effort and trap volume were missing for 50 and 161 occurrences, respectively. We generated accurate sampling effort for 41 occurrences and corrected BIML data for an additional 20. Of the 161 occurrences lacking trap volume, we filled in 47.

## Usage Notes

The BIML data is a rich source of information on wild-bee biodiversity but it has some notable limitations. First, the BIML sampled few locations more than three years. The main goal of BIML collections was to establish baseline estimates of wild-bee species richness in the Mid-Atlantic region. They selected sites representing a wide variety of habitats, focusing on sampling many sites rather than repeated sampling at the same sites over time. Consequently, the BIML dataset has excellent geographic coverage in Maryland, Delaware, and Washington DC (Fig. 1), but lacks long time-series of bee abundance or community composition in consistent locations. Similarly, the BIML did not sample an equal number of sites for all habitat types represented in the dataset. For example, there are relatively few sites in highly agricultural landscapes compared with forested areas, so an analysis of the whole dataset may fail to reveal processes specific to agricultural land. For analyses focused on specific habitat types, we recommend sub-sampling or otherwise adjusting for uneven representation among habitat types.

**Fig. 2:**
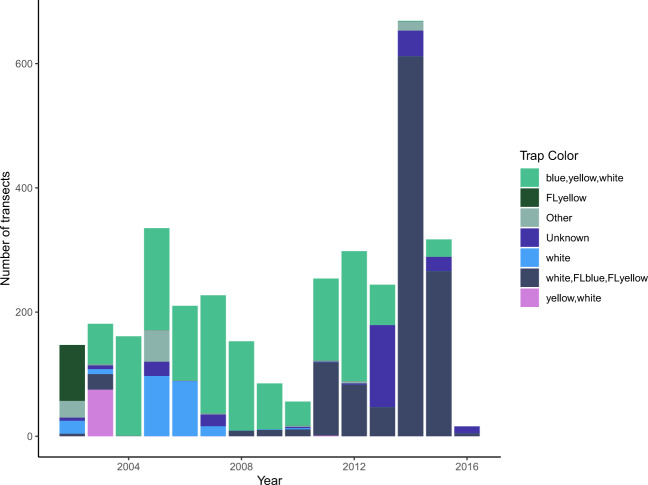
Color of wild bee traps used by USGS BIML from 2002–2016.

**Fig. 3:**
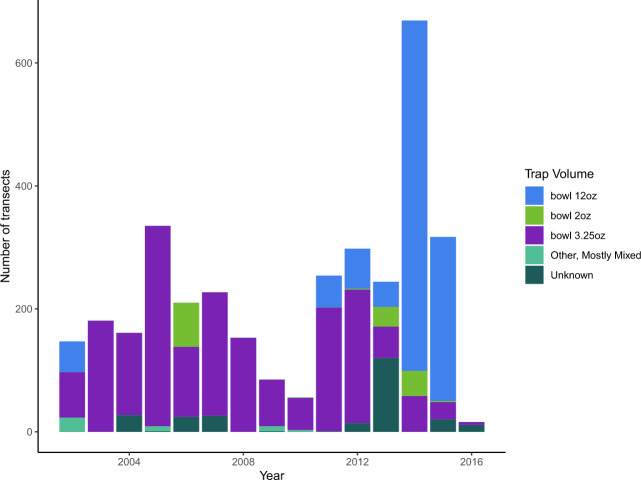
Volume of wild bee traps used by USGS BIML from 2002–2016.

Second, the dominant sampling method changed over the study period. We found that the BIML utilized different colors and volumes of pan traps over time (Figs. 2 and 3). Before 2003, the BIML used a variety of pan-trap colors and volumes. From 2003–2013, the most common sampling method was 3.25 ounce white, blue, and yellow pans, and in 2014, the BIML transitioned almost all their sampling to 12-ounce white, fluorescent blue, and fluorescent yellow bowls.

These features limit, or at best complicate, interpretation of temporal patterns of bee abundance or community composition in the BIML data. For example, a pattern of declining bee abundance over time could be explained by a less effective sampling method or sampling poorer sites or less attractive habitats in later years. In our view, the BIML data are not well suited for assessing temporal trends, as researchers must account for sampling and location changes over time or use a small subset of the data sampled with consistent methodology.

The BIML data we archive here were sampled with pan traps, which impose some inherent biases and limitations. Pan traps significantly under sample large-bodied bees^15,27^, probably because larger-bodied individuals are able to break surface tension in a container of liquid and fly away. Consequently, *Bombus sp., Xylocopa virginica*, and other large-bodied bees are less common in pan-trapped data than net-collected or observational datasets. Additionally, some evidence suggests pan traps perform poorly in areas with highly abundant flowering plants^28–30^, such as field edges adjacent to mass-flowering crops. In proximity to flowering plants, pan traps are less attractive to wild bees than real flowers, leading to an artificially depauperate wild-bee community captured by the pan traps.

Consequently, the BIML datasets we produced are not well suited for studies on large-bodied taxa or in areas with highly abundant flower resources, however these limitations could likely be addressed by combining this dataset with net collections or direct observations. Net collecting has its own limitations of more active time spent per specimen and observer biases, but there are few limitations in common between the two methods. In addition to pan-trapped specimens, the BIML net-collected approximately 16,000 specimens from 2002–2016 in Maryland, Delaware, and Washington DC^18^, but these collections did not necessarily occur at pan-trapping sites. Also, we did not include net-collected occurrences in our data processing pipeline, so species names should be verified before analysis, and sampling effort information may not exist.

We also advise future analysts that some BIML sampling locations are very close to each other. We created ‘site’ designations as all unique combinations of latitude and longitude, no matter the distance from other sites. In the format we provide, the BIML data violates the assumption of independent observations imposed by a frequentist statistical framework. Not all analytical frameworks assume independent observations, so we did not combine or thin sites, but refer interested parties to statistical methods or software designed for this task (k-means clustering or similar, *spThin*^31^ for spatial thinning).

## Supplementary information

Supplementary Information

## Data Availability

All R code used to generate the archived data is available at Zenodo^26^.

## Online-only Tables

**Online-only Table 1:** List of all specimen names that were modified or removed during data cleaning with justification for each change.

Original species name	Cleaned name	Grouped name	Genus	species	Family	Reason for modification/removal
Modified species names						
Andrena arabis/algida	Andrena algida/arabis	Andrena algida/arabis	Andrena	algida/arabis	Andrenidae	Reordered multiple species names
Lasioglossum carlini	Andrena carlini	Andrena carlini	Andrena	carlini	Andrenidae	Incorrect genus
Andrena tridens/erythronii	Andrena erythronii/tridens	Andrena erythronii/tridens	Andrena	erythronii/tridens	Andrenidae	Reordered multiple species names
Amdrema imitatrix/morrisonella	Andrena imitatrix/morrisonella	Andrena imitatrix/morrisonella	Andrena	imitatrix/morrisonella	Andrenidae	Corrected spelling
Andrena morisonella	Andrena morrisonella	Andrena imitatrix/morrisonella	Andrena	morrisonella	Andrenidae	Corrected spelling
Melissodes nivalis	Andrena nivalis	Andrena nivalis	Andrena	nivalis	Andrenidae	Incorrect genus
Lasioglossum perplexa	Andrena perplexa	Andrena perplexa	Andrena	perplexa	Andrenidae	Incorrect genus
Nomada personata	Andrena personata	Andrena personata	Andrena	personata	Andrenidae	Incorrect genus
Andrena puni	Andrena pruni	Andrena pruni	Andrena	pruni	Andrenidae	Corrected spelling
Pseudopanurgus rudbeckiae	Pseudopanurgus near rudbeckiae	Pseudopanurgus near rudbeckiae	Pseudopanurgus	rudbeckiae	Andrenidae	Recommended by data source (S. Droege)
apis mellifera	Apis mellifera	Apis mellifera	Apis	mellifera	Apidae	Standardized format
bombus bimaculatus	Bombus bimaculatus	Bombus bimaculatus	Bombus	bimaculatus	Apidae	Standardized format
ceratina calcarata	Ceratina calcarata	Ceratina calcarata/dupla/mikmaqi	Ceratina	calcarata	Apidae	Standardized format
Ceratina mikmaqi/calcarata	Ceratina calcarata/mikmaqi	Ceratina calcarata/dupla/mikmaqi	Ceratina	calcarata/mikmaqi	Apidae	Reordered multiple species names
ceratina dupla	Ceratina dupla	Ceratina calcarata/dupla/mikmaqi	Ceratina	dupla	Apidae	Standardized format
Ceratina miqmaki	Ceratina mikmaqi	Ceratina calcarata/dupla/mikmaqi	Ceratina	mikmaqi	Apidae	Corrected spelling
Ceratina Strenua	Ceratina strenua	Ceratina strenua	Ceratina	strenua	Apidae	Standardized format
ceratina strenua	Ceratina strenua	Ceratina strenua	Ceratina	strenua	Apidae	Standardized format
Melissodes tineta	Melissodes tinctus	Melissodes tinctus	Melissodes	tinctus	Apidae	Corrected spelling
Nomada sayi/illinoensis	Nomada illinoensis/sayi	Nomada illinoensis/sayi	Nomada	illinoensis/sayi	Apidae	Reordered multiple species names
Nomada sayi/illinoense	Nomada illinoensis/sayi	Nomada illinoensis/sayi	Nomada	illinoensis/sayi	Apidae	Corrected spelling
Nomada sulphurata/luteola	Nomada luteola/sulphurata	Nomada luteola/sulphurata	Nomada	luteola/sulphurata	Apidae	Reordered multiple species names
Nomada luteolodies	Nomada luteoloides	Nomada luteoloides	Nomada	luteoloides	Apidae	Corrected spelling
hylaeus affinis/modestus	Hylaeus affinis/modestus	Hylaeus affinis/modestus	Hylaeus	affinis/modestus	Colletidae	Standardized format
hylaeus ornatus	Hylaeus ornatus	Hylaeus ornatus	Hylaeus	ornatus	Colletidae	Standardized format
Agapostemon angelicus/texanus	Agapostemon texanus	Agapostemon texanus	Agapostemon	angelicus/texanus	Halictidae	One species of set not found in Mid-Atlantic USA
augochlorella aurata	Augochlorella aurata	Augochlorella aurata	Augochlorella	aurata	Halictidae	Standardized format
Augochlora aurata	Augochlorella aurata	Augochlorella aurata	Augochlorella	aurata	Halictidae	Incorrect genus
Lasioglossum aurata	Augochlorella aurata	Augochlorella aurata	Augochlorella	aurata	Halictidae	Incorrect genus
Augochlorellagratiosa	Augochlorella gratiosa	Augochlorella gratiosa	Augochlorella	gratiosa	Halictidae	Standardized format
Augochloropsis Metallica	Augochloropsis metallica	Augochloropsis metallica	Augochloropsis	metallica	Halictidae	Standardized format
Halictus poeyi/ligatus	Halictus ligatus/poeyi	Halictus ligatus/poeyi	Halictus	ligatus/poeyi	Halictidae	Reordered multiple species names
Halitcus poeyi/ligatus	Halictus ligatus/poeyi	Halictus ligatus/poeyi	Halictus	ligatus/poeyi	Halictidae	Corrected spelling
Haclictus tectus	Halictus tectus	Halictus tectus	Halictus	tectus	Halictidae	Corrected spelling
Andrena bruneri	Lasioglossum bruneri	Lasioglossum bruneri	Lasioglossum	bruneri	Halictidae	Incorrect genus
Ceratina callidum	Lasioglossum callidum	Lasioglossum callidum	Lasioglossum	callidum	Halictidae	Incorrect genus
lasioglossum coreopsis	Lasioglossum coreopsis	Lasioglossum coreopsis	Lasioglossum	coreopsis	Halictidae	Standardized format
Lasioglossum coeropsis	Lasioglossum coreopsis	Lasioglossum coreopsis	Lasioglossum	coreopsis	Halictidae	Corrected spelling
Lasioglossum geminum	Lasioglossum geminatum	Lasioglossum geminatum	Lasioglossum	geminatum	Halictidae	Corrected spelling
Lasioglossum Gotham	Lasioglossum gotham	Lasioglossum gotham	Lasioglossum	gotham	Halictidae	Standardized format
Andrena hitchensi	Lasioglossum hitchensi	Lasioglossum hitchensi/weemsi	Lasioglossum	hitchensi	Halictidae	Incorrect genus
Nomada lustrans	Lasioglossum lustrans	Lasioglossum lustrans	Lasioglossum	lustrans	Halictidae	Incorrect genus
lasioglossum pilosum	Lasioglossum pilosum	Lasioglossum pilosum	Lasioglossum	pilosum	Halictidae	Standardized format
Sphecodes cressonii/atlantis	Sphecodes atlantis/cressonii	Sphecodes atlantis/banksii/cressonii	Sphecodes	atlantis/cressonii	Halictidae	Reordered multiple species names
Hoplitis producta/pilosifrons	Hoplitis pilosifrons/producta	Hoplitis pilosifrons/producta	Hoplitis	pilosifrons/producta	Megachilidae	Reordered multiple species names
hoplitis spoliata	Hoplitis spoliata	Hoplitis spoliata	Hoplitis	spoliata	Megachilidae	Standardized format
Megachile apicata	Megachile apicalis	Megachile apicalis	Megachile	apicalis	Megachilidae	Corrected spelling
megachile brevis	Megachile brevis	Megachile brevis/mendica	Megachile	brevis	Megachilidae	Standardized format
Megachile mendica/brevis	Megachile brevis/mendica	Megachile brevis/mendica	Megachile	brevis/mendica	Megachilidae	Reordered multiple species names
Osmia atriventis	Osmia atriventris	Osmia atriventris/pumila	Osmia	atriventris	Megachilidae	Corrected spelling
Osmia pumila/atriventris	Osmia atriventris/pumila	Osmia atriventris/pumila	Osmia	atriventris/pumila	Megachilidae	Reordered multiple species names
Osmia callinsia	Osmia collinsiae	Osmia collinsiae	Osmia	collinsiae	Megachilidae	Corrected spelling
Lasioglossum conjuncta	Osmia conjuncta	Osmia conjuncta	Osmia	conjuncta	Megachilidae	Incorrect genus
Osmia conjucta	Osmia conjuncta	Osmia conjuncta	Osmia	conjuncta	Megachilidae	Corrected spelling
osmia sandhouseae	Osmia sandhouseae	Osmia sandhouseae	Osmia	sandhouseae	Megachilidae	Standardized format
Removed species names						
Lasioglossum melanopus						Not found in Mid-Atlantic USA
Lasioglossum incompletum						Not found in Mid-Atlantic USA
Hylaeus cressonii						Species epithet could not be verified
Lasioglossum kevensi						Species epithet could not be verified
Osmia composita						Species epithet could not be verified
Removed, Genus-only identifications						
Aculeata						Specimen identified to genus or subgenus
Agapostemon						Specimen identified to genus or subgenus
Ancistrocerus						Specimen identified to genus or subgenus
Andrena						Specimen identified to genus or subgenus
Andrena (Melandrena)						Specimen identified to genus or subgenus
Andrena (Scrapteropsis)						Specimen identified to genus or subgenus
Andrena (Trachandrena)						Specimen identified to genus or subgenus
Andrena melandrena						Specimen identified to genus or subgenus
Anthophila						Specimen identified to genus or subgenus
Apidae						Specimen identified to genus or subgenus
Apoidea						Specimen identified to genus or subgenus
Arachnida						Specimen identified to genus or subgenus
Augochlorella						Specimen identified to genus or subgenus
Bombus						Specimen identified to genus or subgenus
Carabidae						Specimen identified to genus or subgenus
Ceratina						Specimen identified to genus or subgenus
Chalcid						Specimen identified to genus or subgenus
Chrysidid						Specimen identified to genus or subgenus
Chrysididae						Specimen identified to genus or subgenus
Coelioxys						Specimen identified to genus or subgenus
Colletes						Specimen identified to genus or subgenus
Figitidae/Eucoilinae						Specimen identified to genus or subgenus
Formicidae						Specimen identified to genus or subgenus
Halictus						Specimen identified to genus or subgenus
Hedychridium						Specimen identified to genus or subgenus
Heriades						Specimen identified to genus or subgenus
Hoplitis						Specimen identified to genus or subgenus
Hylaeus						Specimen identified to genus or subgenus
Ichneumonidae						Specimen identified to genus or subgenus
Ichneumonidae/Ichneunominae						Specimen identified to genus or subgenus
Ichneumonidae/Phygadeuontinae						Specimen identified to genus or subgenus
Ichneumonidae/Tryphoninae						Specimen identified to genus or subgenus
Larrinae						Specimen identified to genus or subgenus
lasioglossum						Specimen identified to genus or subgenus
Lasioglossum						Specimen identified to genus or subgenus
Lasioglossum male						Specimen identified to genus or subgenus
Megachile						Specimen identified to genus or subgenus
Melissodes						Specimen identified to genus or subgenus
Nomada						Specimen identified to genus or subgenus
Osmia						Specimen identified to genus or subgenus
Panurginus						Specimen identified to genus or subgenus
Parancistrocerus						Specimen identified to genus or subgenus
Perdita						Specimen identified to genus or subgenus
Pompilidae						Specimen identified to genus or subgenus
Pseudopanurgus						Specimen identified to genus or subgenus
Pteromalidae						Specimen identified to genus or subgenus
Scoliidae						Specimen identified to genus or subgenus
Sphecidae						Specimen identified to genus or subgenus
sphecodes						Specimen identified to genus or subgenus
Sphecodes						Specimen identified to genus or subgenus
Sphecodogastra						Specimen identified to genus or subgenus
Svastra						Specimen identified to genus or subgenus
Tenthredinidae						Specimen identified to genus or subgenus
Tenthredo						Specimen identified to genus or subgenus
Tiphia						Specimen identified to genus or subgenus
Tiphiidae						Specimen identified to genus or subgenus
Tiphiidae/Tiphiinae						Specimen identified to genus or subgenus
Tiphiinae						Specimen identified to genus or subgenus
Triepeolus						Specimen identified to genus or subgenus
Vespidae						Specimen identified to genus or subgenus
Zanysson						Specimen identified to genus or subgenus
Removed, species that are not bees						
Acmaeodera ornata						Species outside superfamily *Apoidea*, clade *Anthophila*
Acmaeodera pulchella						Species outside superfamily *Apoidea*, clade *Anthophila*
Acmaeodera tubulus						Species outside superfamily *Apoidea*, clade *Anthophila*
Ammophila fernaldi						Species outside superfamily *Apoidea*, clade *Anthophila*
Ammophila nigricans						Species outside superfamily *Apoidea*, clade *Anthophila*
Ammophila pictipennis						Species outside superfamily *Apoidea*, clade *Anthophila*
Ammophila procera						Species outside superfamily *Apoidea*, clade *Anthophila*
Ammophila urnaria						Species outside superfamily *Apoidea*, clade *Anthophila*
Anacrabro ocellatus						Species outside superfamily *Apoidea*, clade *Anthophila*
Ancistrocerus adiabatus						Species outside superfamily *Apoidea*, clade *Anthophila*
Ancistrocerus albophaleratus						Species outside superfamily *Apoidea*, clade *Anthophila*
Ancistrocerus antilope						Species outside superfamily *Apoidea*, clade *Anthophila*
Ancistrocerus campestris						Species outside superfamily *Apoidea*, clade *Anthophila*
Ancistrocerus cressonii						Species outside superfamily *Apoidea*, clade *Anthophila*
Argogorytes nigrifrons						Species outside superfamily *Apoidea*, clade *Anthophila*
Astata unicolor						Species outside superfamily *Apoidea*, clade *Anthophila*
Bicyrtes ventralis						Species outside superfamily *Apoidea*, clade *Anthophila*
Caenochrysis doriae						Species outside superfamily *Apoidea*, clade *Anthophila*
Caenochrysis doriae/Cleptes alienus						Species outside superfamily *Apoidea*, clade *Anthophila*
Ceratochrysis declinis						Species outside superfamily *Apoidea*, clade *Anthophila*
Ceratochrysis enhuycki						Species outside superfamily *Apoidea*, clade *Anthophila*
Ceratochrysis kansensis						Species outside superfamily *Apoidea*, clade *Anthophila*
Chalybion californicum						Species outside superfamily *Apoidea*, clade *Anthophila*
Chrysis angolensis						Species outside superfamily *Apoidea*, clade *Anthophila*
Chrysis antennalis						Species outside superfamily *Apoidea*, clade *Anthophila*
Chrysis cembricola						Species outside superfamily *Apoidea*, clade *Anthophila*
Chrysis conica						Species outside superfamily *Apoidea*, clade *Anthophila*
Chrysis derivata						Species outside superfamily *Apoidea*, clade *Anthophila*
Chrysis inaequidens						Species outside superfamily *Apoidea*, clade *Anthophila*
Chrysis montana						Species outside superfamily *Apoidea*, clade *Anthophila*
Chrysis nisseri						Species outside superfamily *Apoidea*, clade *Anthophila*
Chrysis nitidula/coerulans						Species outside superfamily *Apoidea*, clade *Anthophila*
Chrysis pattoni						Species outside superfamily *Apoidea*, clade *Anthophila*
Chrysis pellucidula						Species outside superfamily *Apoidea*, clade *Anthophila*
Chrysis propria						Species outside superfamily *Apoidea*, clade *Anthophila*
Chrysis remissa						Species outside superfamily *Apoidea*, clade *Anthophila*
Chrysis scitula						Species outside superfamily *Apoidea*, clade *Anthophila*
Chrysis smaragdala						Species outside superfamily *Apoidea*, clade *Anthophila*
Chrysis tripartita						Species outside superfamily *Apoidea*, clade *Anthophila*
Chrysura cobaltina						Species outside superfamily *Apoidea*, clade *Anthophila*
Chrysura cobaltina/pacifica						Species outside superfamily *Apoidea*, clade *Anthophila*
Chrysura kyrae/pacifica						Species outside superfamily *Apoidea*, clade *Anthophila*
Chrysura pacifica						Species outside superfamily *Apoidea*, clade *Anthophila*
Cicindella sexguttata						Species outside superfamily *Apoidea*, clade *Anthophila*
Cinthophora abrupta						Species outside superfamily *Apoidea*, clade *Anthophila*
Compsomeris plumipes						Species outside superfamily *Apoidea*, clade *Anthophila*
Dolichovespula arenaria						Species outside superfamily *Apoidea*, clade *Anthophila*
Dolichovespula maculata						Species outside superfamily *Apoidea*, clade *Anthophila*
Ectemnius lapidarius						Species outside superfamily *Apoidea*, clade *Anthophila*
Ectemnius rufipes						Species outside superfamily *Apoidea*, clade *Anthophila*
Ectemnius scaber						Species outside superfamily *Apoidea*, clade *Anthophila*
Epinysson mellipes						Species outside superfamily *Apoidea*, clade *Anthophila*
Eremnophila aureonotata						Species outside superfamily *Apoidea*, clade *Anthophila*
Eumenes fraternus						Species outside superfamily *Apoidea*, clade *Anthophila*
Eumenes reticalis						Species outside superfamily *Apoidea*, clade *Anthophila*
Euodynerus annulatus						Species outside superfamily *Apoidea*, clade *Anthophila*
Euodynerus boscii						Species outside superfamily *Apoidea*, clade *Anthophila*
Euodynerus castigatus						Species outside superfamily *Apoidea*, clade *Anthophila*
Euodynerus crypticus						Species outside superfamily *Apoidea*, clade *Anthophila*
Euodynerus foraminatus						Species outside superfamily *Apoidea*, clade *Anthophila*
Euodynerus hidalgo						Species outside superfamily *Apoidea*, clade *Anthophila*
Euodynerus hidalgo boreoorientalis						Species outside superfamily *Apoidea*, clade *Anthophila*
Euodynerus leucomelas						Species outside superfamily *Apoidea*, clade *Anthophila*
Euodynerus megaera						Species outside superfamily *Apoidea*, clade *Anthophila*
Euodynerus schwarzi						Species outside superfamily *Apoidea*, clade *Anthophila*
Euodyrnus megaera						Species outside superfamily *Apoidea*, clade *Anthophila*
Hedychridium dimidiatum						Species outside superfamily *Apoidea*, clade *Anthophila*
Hedychrum confusum/violaceum						Species outside superfamily *Apoidea*, clade *Anthophila*
Hedychrum parvum						Species outside superfamily *Apoidea*, clade *Anthophila*
Hedychrum violaceum						Species outside superfamily *Apoidea*, clade *Anthophila*
Isodontia apicalis						Species outside superfamily *Apoidea*, clade *Anthophila*
Isodontia auripes						Species outside superfamily *Apoidea*, clade *Anthophila*
Isodontia mexicana						Species outside superfamily *Apoidea*, clade *Anthophila*
Isodontia philadelphica						Species outside superfamily *Apoidea*, clade *Anthophila*
Leptochilus acolhuus						Species outside superfamily *Apoidea*, clade *Anthophila*
Leptochilus republicans						Species outside superfamily *Apoidea*, clade *Anthophila*
Leptochilus republicanus						Species outside superfamily *Apoidea*, clade *Anthophila*
Lestica confluenta						Species outside superfamily *Apoidea*, clade *Anthophila*
Lestica producticollis						Species outside superfamily *Apoidea*, clade *Anthophila*
Liris argentatus						Species outside superfamily *Apoidea*, clade *Anthophila*
Lyroda subita						Species outside superfamily *Apoidea*, clade *Anthophila*
Monobia quadridens						Species outside superfamily *Apoidea*, clade *Anthophila*
Oxybelus emarginatum						Species outside superfamily *Apoidea*, clade *Anthophila*
Oxybelus emarginatus						Species outside superfamily *Apoidea*, clade *Anthophila*
Oxybelus subulatus						Species outside superfamily *Apoidea*, clade *Anthophila*
Palmodes collinsiae						Species outside superfamily *Apoidea*, clade *Anthophila*
Palmodes dimidiatus						Species outside superfamily *Apoidea*, clade *Anthophila*
Palmodes pumila						Species outside superfamily *Apoidea*, clade *Anthophila*
Parancistrocerus fulvipes						Species outside superfamily *Apoidea*, clade *Anthophila*
Parancistrocerus histrio						Species outside superfamily *Apoidea*, clade *Anthophila*
Parancistrocerus leionotus						Species outside superfamily *Apoidea*, clade *Anthophila*
Parancistrocerus pedestris						Species outside superfamily *Apoidea*, clade *Anthophila*
Parancistrocerus pedestris or pensylvanicus						Species outside superfamily *Apoidea*, clade *Anthophila*
Parancistrocerus pedestris pedestris						Species outside superfamily *Apoidea*, clade *Anthophila*
Parancistrocerus pensylvanicus						Species outside superfamily *Apoidea*, clade *Anthophila*
Parancistrocerus perennis						Species outside superfamily *Apoidea*, clade *Anthophila*
Parancistrocerus salcularis						Species outside superfamily *Apoidea*, clade *Anthophila*
Parancistrocerus vagus						Species outside superfamily *Apoidea*, clade *Anthophila*
Parazumia perennis						Species outside superfamily *Apoidea*, clade *Anthophila*
Parazumia symmorpha						Species outside superfamily *Apoidea*, clade *Anthophila*
Philanthus gibbosus						Species outside superfamily *Apoidea*, clade *Anthophila*
Polistes bellicosus						Species outside superfamily *Apoidea*, clade *Anthophila*
Polistes dominula						Species outside superfamily *Apoidea*, clade *Anthophila*
Polistes dorsalis						Species outside superfamily *Apoidea*, clade *Anthophila*
Polistes exclamans						Species outside superfamily *Apoidea*, clade *Anthophila*
Polistes fuscatus						Species outside superfamily *Apoidea*, clade *Anthophila*
Polistes hirsuticornis						Species outside superfamily *Apoidea*, clade *Anthophila*
Polistes metricus						Species outside superfamily *Apoidea*, clade *Anthophila*
Polistes parametricus						Species outside superfamily *Apoidea*, clade *Anthophila*
Pompilidae Ctenocerinae						Species outside superfamily *Apoidea*, clade *Anthophila*
Prionyx parkeri						Species outside superfamily *Apoidea*, clade *Anthophila*
Pseduoanthidium nanum						Species outside superfamily *Apoidea*, clade *Anthophila*
Pseudodynerus quadrisectus						Species outside superfamily *Apoidea*, clade *Anthophila*
Scolia dubia						Species outside superfamily *Apoidea*, clade *Anthophila*
Sphex nudus						Species outside superfamily *Apoidea*, clade *Anthophila*
Sphex pensylvanicus						Species outside superfamily *Apoidea*, clade *Anthophila*
Stenodynerus ammonia						Species outside superfamily *Apoidea*, clade *Anthophila*
Stenodynerus ammonia paraensis						Species outside superfamily *Apoidea*, clade *Anthophila*
Stenodynerus anormis						Species outside superfamily *Apoidea*, clade *Anthophila*
Stenodynerus blepharus						Species outside superfamily *Apoidea*, clade *Anthophila*
Stenodynerus fundatiformis						Species outside superfamily *Apoidea*, clade *Anthophila*
Stenodynerus fundatiformis gonosceles						Species outside superfamily *Apoidea*, clade *Anthophila*
Stenodynerus histrionalis						Species outside superfamily *Apoidea*, clade *Anthophila*
Stenodynerus krombeini						Species outside superfamily *Apoidea*, clade *Anthophila*
Stenodynerus oculeus						Species outside superfamily *Apoidea*, clade *Anthophila*
Symmorphus canadensis						Species outside superfamily *Apoidea*, clade *Anthophila*
Synnevrus plagiatus						Species outside superfamily *Apoidea*, clade *Anthophila*
Tachysphex acutus						Species outside superfamily *Apoidea*, clade *Anthophila*
Tachysphex antennatus						Species outside superfamily *Apoidea*, clade *Anthophila*
Tachysphex belfragei						Species outside superfamily *Apoidea*, clade *Anthophila*
Tachysphex mundus						Species outside superfamily *Apoidea*, clade *Anthophila*
Tachysphex similis						Species outside superfamily *Apoidea*, clade *Anthophila*
Tachysphex tarsatus						Species outside superfamily *Apoidea*, clade *Anthophila*
Tachysphex terminatus						Species outside superfamily *Apoidea*, clade *Anthophila*
Tachytes abdominalis						Species outside superfamily *Apoidea*, clade *Anthophila*
Tachytes chrysopyga obscurus						Species outside superfamily *Apoidea*, clade *Anthophila*
Tachytes distinctus						Species outside superfamily *Apoidea*, clade *Anthophila*
Tachytes guatemalensis						Species outside superfamily *Apoidea*, clade *Anthophila*
Tachytes validus						Species outside superfamily *Apoidea*, clade *Anthophila*
Trypoxylon clavicerum						Species outside superfamily *Apoidea*, clade *Anthophila*
Trypoxylon frigidum						Species outside superfamily *Apoidea*, clade *Anthophila*
Trypoxylon pennsylvanicum						Species outside superfamily *Apoidea*, clade *Anthophila*
Typocerus velutinus						Species outside superfamily *Apoidea*, clade *Anthophila*
Vespa crabro						Species outside superfamily *Apoidea*, clade *Anthophila*
Vespula consobrina						Species outside superfamily *Apoidea*, clade *Anthophila*
Vespula flavopilosa						Species outside superfamily *Apoidea*, clade *Anthophila*
Vespula germanica						Species outside superfamily *Apoidea*, clade *Anthophila*
Vespula maculifrons						Species outside superfamily *Apoidea*, clade *Anthophila*
Vespula squamosa						Species outside superfamily *Apoidea*, clade *Anthophila*
Vespula vidua						Species outside superfamily *Apoidea*, clade *Anthophila*
Zethus spinipes						Species outside superfamily *Apoidea*, clade *Anthophila*
Zethus spinipes variegatus						Species outside superfamily *Apoidea*, clade *Anthophila*

Species names included in the final datasets^22^ were verified against a current species list^23^ and reviewed by S. Droege, the original data producer.

**Online-only Table 2:** Description of all fields in archived dataset 2, wild bee specimens in Maryland, Delaware, and Washington DC with recorded sampling effort.

Field Name	Unit	Description
Identifiers		
identifier		Original, specimen ID
id		Original, specimen URL
TransectID		transect ID
SamplEvent		sampling event ID
SiteID		site ID
SiteID_Year		site ID and year of bee sampling
year		year of bee sampling
Taxonomy		
name		taxonomic classification
Genus		taxonomic classification
species		taxonomic classification
grouped_name		grouped taxonomic classification
orig_name		Original, taxonomic classification
sex		Original
identifiedBy		Individual who determined specimen identity
Locality and Date		
latitude	decimal degrees	Original
longitude	decimal degrees	Original
coordinateUncertaintyInMeters	meters	Original
time1		Original, start date and time
time2		Original, end date and time
startdate	month/day/year	sampling start date
enddate	month/day/year	sampling end date
trapdays	days	length of sampling period
country		Original
countryCode		Original
state		Original
county		Original
municipality		Original
Field notes		
habitat		Original, habitat in location of sampling
field_note		Original
note		Original
modif_fieldnote		modified field note used for automated string search
modif_note		modified note used for automated string search
Sampling method and effort		
SampleType		type of sampling method
TrapLiquid		Original, liquid used in traps
TrapColor		Original, color of trap
TrapVolume		Original, volume of trap
Ntraps		Original, number of traps
nwhite		number of white pan traps
nblue		number of blue pan traps
nyellow		number of yellow pan traps
nFLblue		number of fluorescent blue pan traps
nFLyellow		number of fluorescent yellow pan traps
nFLwhite		number of fluorescent white pan traps
isblue	T/F	sampling method = blue pan traps?
isyellow	T/F	sampling method = yellow pan traps?
iswhite	T/F	sampling method = white pan traps?
isFLblue	T/F	sampling method = fluorescent blue pan traps?
isFLyellow	T/F	sampling method = fluorescent yellow pan traps?
isFLwhite	T/F	sampling method = fluorescent white pan traps?
ispaleblue	T/F	sampling method = pale blue pan traps?
isFLpaleblue	T/F	sampling method = fluorescent pale blue pan traps?
TrapVolumeParsed		trap volume (from regex string search)
TrapVolumeFinal		trap volume (combined original and regex)*
Nparse		number of traps set (from regex string search)
Nmissing		number of traps missing (from regex string search)
NTrapsFinal		number of successful traps (set-missing)*
Wild bee abundance		
Abundance	specimens	wild bee abundance
AbundDay	specimens day^−1^	wild bee abundance day-1
AbundTrap	specimens trap^−1^	wild bee abundance trap-1
AbundDayTrap	specimens day^−1^ trap^−1^	wild bee abundance day-1 trap-1*

Variables marked as ‘Original’ are unmodified from Droege and Sellers^18^. *Variable recommended for analysis (within set of similar variables).
