# Supplementary material for: A long-term dataset on wild bee abundance in Mid-Atlantic United States
Source: Sci Data. 2020 Jul 20;7:240. doi: 10.1038/s41597-020-00577-0 (PMC7371858; doi:10.1038/s41597-020-00577-0)
Supplement: Supplementary file 1 — Supplementary Information [file 41597_2020_577_MOESM1_ESM.pdf]

Supplementary Information

**identifiedBy**	**Full name**	**Affiliation**
C. Stragar	Catherine Stragar	Plant Protection and Weed Management, Maryland Department of Agriculture
E. Scarpulla	Eugene J. Scarpulla	Maryland Entomological Society and USGS BIML, Patuxent Wildlife Research Center
H. Harmon	Heather Harmon Disque	Forest Pest Management, Maryland Department of Agriculture
J. Ascher	John S. Ascher	Department of Biological Sciences, National University of Singapore
J. Carpenter	James M. Carpenter	Division of Invertebrate Zoology, American Museum of Natural History
J. Devalez	Jelle Devalez	Department of Geography, University of the Aegean
J. Gibbs	Jason Gibbs	Department of Entomology, University of Manitoba
K. Wright	Karen Wright	Department of Entomology, Texas A&M University
M. Arduser	Michael Arduser	Missouri Department of Conversation
M. Orr	Michael Orr	Institute of Zoology, Chinese Academy of Sciences
M. Rightmyer	Molly Rightmyer Gee	San Diego Natural History Museum
S. Droege	Sam Droege	USGS BIML, Patuxent Wildlife Research Center
S. Rehan	Sandra Rehan	Department of Biology, York University
T. McMahon	Tim McMahon	Montgomery County Maryland Beekeepers Association and USGS BIML, Patuxent Wildlife Research Center

Table S1: Full names and affiliations of individuals who identified specimens in the United States Geological Survey Native Bee Inventory and Monitoring Lab dataset.
